# Dietary Bioactives: Their Role in the Prevention and Treatment of Cardiovascular and Metabolic Bone Diseases

**DOI:** 10.3390/nu14122459

**Published:** 2022-06-14

**Authors:** Domitilla Mandatori, Assunta Pandolfi

**Affiliations:** Center for Advanced Studies and Technology—CAST, StemTeCh Group, Department of Medical, Oral and Biotechnological Sciences, University “G. d’Annunzio” of Chieti-Pescara, 66100 Chieti, Italy; assunta.pandolfi@unich.it

Cardiovascular and metabolic bone diseases are demanding health problems with high morbidity and mortality [1,2]. Although for many years, the development of such diseases was considered to be solely age-related [3], evidence has provided support for a close correlation between bone and vascular health [4]. This link, commonly defined “bone-vascular crosstalk” [5], occurs due to the onset of shared molecular and cellular mechanisms to cardiovascular and metabolic bone diseases [6]. Thus, several therapeutical approaches have been proposed to manage these age-related diseases. Among these, there was growing interest in the use of dietary bioactive compounds, which showed promising effects on bone and vascular health. In fact, despite being a highly specific field of study, when the key words “bioactive compounds”, “cardiovascular health” and “bone health” are combined in PubMed, there is a clear increase in published papers in recent years (Figure 1).

In this context, the Special Issue (SI) “Dietary Bioactives: Their Role in the Prevention and Treatment of Cardiovascular and Metabolic Bone Diseases” has published nine novel papers on this topic [7,8,9,10,11,12,13,14,15]. In detail, the SI includes: one narrative review paper, one cross-sectional analysis, three pre-clinical animal studies, three in vitro experimental approaches and one ex vivo approach.

The narrative review was published by Mandatori et al. (2021). The authors summarized the most relevant recent knowledge concerning the role of Vitamin K2, a bioactive compound with a key role in the “calcium paradox” phenomenon, which involves both vascular and bone tissue [16]. The characteristics of this promising natural molecule, its molecular mechanism and clinical outcomes obtained both in bone and vascular disorders were reported in this review [15].

Subsequently, the specific mechanism of Vitamin K2 in bone health was also addressed in an in vitro study [7]. Specifically, the efficacy of Vitamin K2 in improving the functions of osteoblasts isolated from osteoporotic patients was demonstrated. Notably, in this paper, the authors developed innovative 3D bone constructs with the aim of reproducing in vitro, for each osteoporotic patients, the bone remodeling unit composed of the autologous bone cells. An anti-osteoporotic effect was also shown by the bioactive constituents from *Lycii radicis* cortex in a paper published by Park et al. (2021). Using an animal model of ovariectomized-induced osteoporotic mice, the authors identified scopolin as the candidate bioactive compound extracted from *Lycii radicis* cortex capable of preventing and treating osteoporosis [13]. Finally, in the bone health field, the pro-osteogenic effects of the extracted from *Cucurbita moschata* leaves, a pumpkin cultivar in Western countries, were published by Lambertini et al. [8].

Regarding cardiovascular health, in this Special Issue, one in vitro, one ex vivo and two pre-clinical animal studies were published. Baldassare et al. (2021) reported the anti-inflammatory and anti-oxidative role of myo-inositol using a model of cultured human endothelial cells isolated from the umbilical cord vein of women affected by gestational diabetes [10]. Indeed, these cells being exposed to chronic hyperglycemia in vivo during pregnancy, show a typical pro-inflammatory and pro-oxidative phenotype representing a suitable model for the study of vascular dysfunction [17]. Anti-inflammatory and anti-oxidative properties were also shown by the *Allium sativum* extract in an ex vivo study on mouse heart samples exposed to *E. coli* lipopolysaccharide inflammatory stimulus [9]. Additionally, the two pre-clinical animal studies highlighted—(*i*) the capability of *Sasa quelpaertensis* to ameliorate metabolic dysfunction conditions including dyslipidemia, insulin resistance, and hepatic lipid accumulation, induce in rats by a high-fructose-diet [12] and (*ii*) the protective effects of *Vitis labrusca* on cardiovascular dysfunction due to hypertensive conditions—employed the model of Spontaneously Hypertensive Rats [14].

Finally, in this SI, Esposito et al. (2021) published a cross-sectional analysis performed on a sub-cohort of 4592 subjects from the Moli-sani Study (2005–2010) which suggested that intake polyphenols, which contribute to slowing down the biological aging process, may exert protective effects on the long-term risk of cardiovascular and metabolic bone disease development [11].

In conclusion, this SI allowed us to publish a number of encouraging scientific studies based on in vitro, ex vivo and in vivo approaches confirming the increasing interest of researchers in the discovery of new potential bioactive compounds for human health. However, future research must better understand the mechanisms of action of natural molecules and nutritional supplements for the management of cardiovascular and metabolic bone diseases.

## Figures and Tables

**Figure 1:**
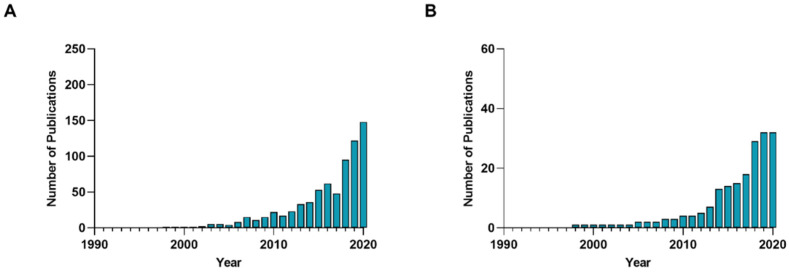
Number of published publications focused on the role of bioactive compound in (**A**) cardiovascular and (**B**) bone health. “Bioactive compounds for cardiovascular health” or “Bioactive compounds for bone health” were the key words used for PubMed searching analyses.

## Data Availability

Not applicable.
